# Self-Consistent Parameterization of DNA Residues for the Non-Polarizable AMBER Force Fields

**DOI:** 10.3390/life12050666

**Published:** 2022-04-30

**Authors:** Amelia L. Schneider, Amanda V. Albrecht, Kenneth Huang, Markus W. Germann, Gregory M. K. Poon

**Affiliations:** 1 Department of Chemistry, Georgia State University, Atlanta, GA 30303, USA; aschneider@gsu.edu (A.L.S.); aalbrecht1@gsu.edu (A.V.A.); khuang8@gsu.edu (K.H.); 2 Department of Biology, Georgia State University, Atlanta, GA 30303, USA; 3 Center for Diagnostics and Therapeutics, Georgia State University, Atlanta, GA 30303, USA

**Keywords:** nucleic acids, DNA, charge, electrostatic potential, ab initio methods, NMR spectroscopy, molecular dynamics, forcefield, AMBER

## Abstract

Fixed-charge (non-polarizable) forcefields are accurate and computationally efficient tools for modeling the molecular dynamics of nucleic acid polymers, particularly DNA, well into the µs timescale. The continued utility of these forcefields depends in part on expanding the residue set in step with advancing nucleic acid chemistry and biology. A key step in parameterizing new residues is charge derivation which is self-consistent with the existing residues. As atomic charges are derived by fitting against molecular electrostatic potentials, appropriate structural models are critical. Benchmarking against the existing charge set used in current AMBER nucleic acid forcefields, we report that quantum mechanical models of deoxynucleosides, even at a high level of theory, are not optimal structures for charge derivation. Instead, structures from molecular mechanics minimization yield charges with up to 6-fold lower RMS deviation from the published values, due to the choice of such an approach in the derivation of the original charge set. We present a contemporary protocol for rendering self-consistent charges as well as optimized charges for a panel of nine non-canonical residues that will permit comparison with literature as well as studying the dynamics of novel DNA polymers.

## 1. Introduction

The commercial success of consumer-grade graphics processing units (GPUs) and their adoption by major molecular dynamics (MD) packages has rendered practical many atomistic explicit-solvent simulations on affordable commodity computers. In the case of DNA, the AMBER forcefield continues to enjoy widespread use more than twenty years since the release of the second generation by Cornell et al. [1]. This popularity is attributable to subsequent reparameterization of the parameter set (parm94) that captures dynamic behavior to the µs timescale [2,3,4,5]. These changes (the latest known as OL15 [6] and parmbsc1 [7] for DNA) have involved the complete parametrization of the backbone dihedral potentials while retaining the atomic charges in parm94. This evolution contrasts with the extensive reparameterization of the charge set for proteins post-ff94 [8]. There is consensus that, taken together, these refinements in OL15 and parmbsc1 represent the accuracy limits for classical DNA forcefields based on fixed-charge two-body interactions [2]. Efforts are underway to overcome the limitations of classical forcefields, such as by incorporating nuclear quantum effects in so-called ab initio MD or AIMD [9,10,11]. Currently, the computational demands of AIMD mostly limit its application to the detailed solvation chemistry of low-MW systems over the fs-ps timescale [12]. Interrogation of biomolecular polymers exhibiting ns-µs timescales dynamics, which for many purposes do not require quantum mechanical treatment, remain very amenable to classical forcefields. One may therefore expect continued utility of AMBER forcefields in molecular mechanics work of DNA for the foreseeable future.

The derivation of atomic charges is critical to correctly capture noncovalent interactions in a classical forcefield. For the AMBER series of biomolecular forcefields, atomic charges are fundamentally derived from fitting against a quantum mechanical (QM) model of the electrostatic potential (ESPs) at the molecular surface [13]. For parm94, ESP-fitted charges are computed using the 6-31G* basis set in the gas phase [1]. The 6-31G* basis set, which is known to overestimate bond polarity, is chosen deliberately for condensed-phase systems to balance water models (such as TIP3P and TIP4P) which are themselves hyperpolarized over the gas-phase value for water [14]. Error compensation in water-solute and water-water interactions is an inherent feature of fixed-charge forcefields that lack accounting polarizability and nuclear quantum effects [9,10]. To mitigate spurious sensitivity of ESP fitting to molecular conformation, a second model, known as restrained electrostatic potential (RESP) [15], was devised to “restrain” polarization of buried atoms, which are poorly determined by surface ESPs, towards a zero value during the fit. RESP fitting is a key finishing step in the parameterization of novel solutes [16] as well as building blocks for nucleic acids, proteins, and carbohydrates [17].

For polymeric solutes that exemplify biomolecular macromolecules such as nucleic acids, it is critical that all the residues be parameterized on an equivalent, self-consistent basis. Self-consistency is a specific concern when new residues are introduced and incorporated with existing residues in a mixed polymer. The original set of nucleic acid residues in AMBER contains only the canonical set of A, C, G, and T/U. Advances in solid-state phosphoramidite chemistry have greatly broadened the scope of nucleic acid residues, many of which have been parameterized for the AMBER forcefield. For DNA, they include non-canonical bases that occur naturally, such as hypoxanthine, epigenetically modified cytosines (e.g., 5-methylcytosine), diaminopurine (DAP or 2-aminoadenine, 2AA), and DNA damage products (e.g., 8-oxoguanine). In addition, many non-natural nucleobases, such as 2-aminopurine, are used as spectroscopic and chemical probes in molecular biology. Given that mixed sequences of new and canonical residues are typical, it is clearly of interest to parameterize novel residues to preserve self-consistency with the original canonical bases.

From a self-consistency perspective, RESP fitting is a critical step because it allows for globally fitting multiple species, with flexibility in fixing, sharing, and restricting charge assignments during the fit [18]. In the parm94 nucleic acids charge set, which were RESP fitted from ESPs computed at the HF/6-31G* level, values for the backbone (deoxyribose and phosphate) atoms are shared across the four canonical bases, with the exception of the C1’ and H1’ atoms. The latter two atoms float with the variable nucleobase atoms during the fit. In the literature, parameterization of new residues has generally adhered to this scheme. More critical and unfortunately less uniform, however, are the structures used to derive the ESP and RESP-fitted charges. Because atomic charges are fitted against the surface molecular potential, ESP fitting is highly sensitive to molecular conformation [15]. Although RESP is more robust to the statistical ill effects of buried atoms than ESP fitting [19], conformational effects on the derived atomic charges are general and reflect the molecular microenvironment. The need to control for conformational effects on charge derivation has spurred several innovations, such as the R.E.D. tools by Dupradeau, Cieplak, and coworkers [20], aimed at standardizing and automating the charge derivation workflow.

In the original charge derivation of the canonical nucleic acids in parm94 [18], structures were derived by molecular mechanics (MM) minimization using the previous-generation ff86 forcefield by Weiner et al. [21,22]. This choice was presumably due to the computational demand of the time for ab initio optimization of whole nucleosides. In contrast, contemporary charge parameterization typically begins with geometry optimization of de novo models at the HF/6-31G* level which is affordable nowadays [23]. In principle, QM optimization should yield physically more accurate structures, but the consistency of this contemporary practice with the parm94 charge set is not obvious and has never been clarified to our knowledge. If the QM-optimized structures do not sufficiently capture the peculiarity of the MM models (however flawed the latter may be relative to the former), the self-consistency of the forcefield with respect to a mixed polymer could be compromised.

How could this be tested? A major stated design principle of the AMBER forcefield is transferability. Adhering to this principle, factors that impact the parameterization of a new residue should similarly impact the canonical residues, whose RESP-fitted charges are known, i.e., parm94. An unambiguous approach to testing the self-consistency of a parameterization protocol with the forcefield is therefore to apply the protocol to extant residues in parm94. If the protocol is self-consistent with the derivation of ff94, it should naturally reproduce the atomic charges of the original bases in the forcefield. The purpose of this work is two-fold. (1) Determine a parameterization protocol for ff94 that best preserves self-consistency with the canonical bases in the forcefield. (2) Provide self-consistent parameters for a panel of non-canonical nucleobases, including several that are not yet reported. Here, we concentrate on DNA, but we expect the resultant principles and recommendations to apply to the parameterization of RNA residues as well.

## 2. Materials and Methods

*Chemical structure optimizations*. Initial atomic models were obtained from the ff86 forcefield or generated with GaussView (Version 5.0.9; Gaussian, Wallingford, CT, USA). Coordinates were parameterized to reflect point group symmetry, planarity, or specific conformations as described in the text. Geometry optimization and subsequent quantum mechanical calculations were performed in internal coordinates with Gaussian 16 (Revision A.03; Gaussian). The stationarity of the optimized structures was confirmed with a frequency calculation. MM energy minimization was performed in either AMBER5 or AMBER16 using the *sander* module.

*Atomic charge fitting*. Fitting to a QM electrostatic potential was performed on QM-optimized or otherwise specified structures with Gaussian 16 using the Merz–Singh–Kollman scheme (pop = MK). The ESP was computed at 4 layers (1.4, 1.6, 1.8, 2.0 × the van der Waals radius) and a nominal density of 1 point/Å^2^ [13]. RESP fitting was performed per the reported two-step multi-molecular procedure [18] as described in Supplemental Methods.

*NMR spectroscopy*. Hairpin-forming oligodeoxynucleotides were synthesized by Integrated DNA Technologies (Coralville, IA, USA) by standard phosphoramidite synthesis. The DNA was adjusted to 0.5 M NaCl to dissociate ionic contaminants and purified by size-exclusion chromatography on a 5 × 5 mL HiTrap Desalting column on an ÄKTA instrument (Cytiva, Marlborough, MA, USA) The desalted DNA was lyophilized and dissolved in 20 mM NaH_2_PO_4_/Na_2_HPO_4_ containing 50 mM NaCl and 0.5 mM EDTA. D_2_O was added to 10% and the pH was adjusted to 6.40. NMR experiments were performed on a Bruker Avance I 500 spectrometer equipped with a TBI ^1^H{^13^C, X} z gradient probe. For monitoring imino proton resonances, a 1-1 jump and return sequence was used to record spectra from 288 K to 308 K. Phase-sensitive 1-1 jump and return NOESYs were collected at 288 K with 2048 × 800 data points in the two dimensions and 72 scans per t_1_ increment using a 150 ms mixing time and a 1.0 s relaxation delay. Two-dimensional (2D) spectra were strip transformed and processed using a 4K × 2K matrix. Both dimensions were apodized with shifted sin(π/2) bell functions. Proton chemical shifts were referenced to internal 2,2-dimethyl-2-silapentane-5-sulfonate (DSS).

*Molecular dynamics simulations*. The conformations of an 18-nucleotide (nt) DNA hairpin designed to probe the effect of an internally positioned residue were sampled using the GROMACS 2022 package. The parmbsc1 update [7] of the ff94 forcefield was used. Following topology generation, each system was set up in dodecahedral boxes 1.0 nm wider than the longest dimension of the solute, solvated with TIP3P water, and neutralized with Na^+^ and Cl^−^ to 0.05 M. Electrostatic interactions were handled by the P3M method [24] with a 1 nm distance cutoff. A timestep of 2 fs was used and bonds including hydrogens were constrained using LINCS. After the structures were energy-minimized, the *NVT* ensemble was equilibrated at 298 K (modified Berendsen thermostat) [25] for 1 ns to thermalize the system, followed by another 1 ns of equilibration of the *NPT* ensemble at 1 bar (stochastic cell rescaling) [26] and 298 K. The NPT ensemble was simulated at 298 K without restraints for 200 ns.

*Computational analysis*. Furanose ring puckering was computed according to the Cremer–Pople scheme [27] from ordered atomic coordinates (O4’→ C1’→ C2’→ C3’→ C4’) as described by Chan et al. [28]. For NMR chemical shift calculations, averaged structures were first optimized by energy minimization against parmbsc1 in TIP3P water with a nominal complement of 0.05 M Na^+^ and Cl^−^ ions. Single-point calculations were performed using the GIAO method at the B3LYP/6-31G* level in implicit CPCM water. A calculation on the DSS anion, optimized at B3LYP/6-31 + G*, was performed to reference the computed isotropic shielding tensors.

## 3. Results and Discussion

*MM-minimized structures are superior to QM-optimized models for ff94 charge parameterization*. To determine whether QM-optimized models represented good structures for ff94 parameterization, we optimized the four canonical deoxynucleosides (DAN, DGN, DCN, and DTN) at HF/6-31G*. Optimizations were constrained only to enforce planarity of the purine or pyrimidine rings and a *trans* conformation for the 5’- and 3’-OH relative to the connected heavy atoms, the latter as indicated in the derivation of ff94 [18]. The resultant structures differed from the reported MM-optimized geometry [18], which was provided in summary form in terms of the sugar pucker, the backbone dihedral γ (O5’-C5’-C4’-C3’), and the *N*-glycosidic dihedral χ (O4’-C1’-N9-C4 for purines, O4’-C1’-N1-C2 for pyrimidines), by ~10% (Table 1).

For comparison, we optimized a parallel set of structures at the same level of theory and basis set with additional constraints imposed to match the reported geometry. We note that these geometric parameters do not specify an explicit structure due to the specification of sugar puckers as amplitude *q* and phase *W* according to the convention of Cremer and Pople [27]. Since the Cremer–Pople convention takes directly as input ordered atomic coordinates (O4’→ C1’→ C2’→ C3’→ C4’), the pucker parameters cannot be constrained directly in terms of functions of internal coordinates during optimization. By scanning the endocyclic dihedrals, we determined representative geometries matching the reported phases and amplitudes to within *W* < 0.01 Å (<3% deviation) in phase and *q* < 1° (<0.7%) in amplitude. These ring dihedrals were then additionally constrained during optimization to yield a set of structures in much better agreement (~1% deviation) with the reference geometries.

To assess the impact of these discrepancies on the final atomic charges, we followed the originally described two-step multi-molecular RESP procedure (see Supplemental Methods) [18]. As our immediate objective is to determine how well the structures reproduce the parm94 charges, we did not fix any of the charges, only equivalencing rotatable hydrogen atoms and imposing targeted constraints in combining nucleosides and the phosphate analog (dimethylphosphate) as specified by Cieplak et al. [18]. The imposition of puckering and *N*-glycosidic geometries reported in Cieplak et al., as could be practically executed, yielded final RESP charge parameters in better agreement with parm94 values, albeit less than expected from the improved agreement in geometry (Table S1, Supplementary Materials). To test if agreement might be further improved with more polished structures, we fitted nucleosides optimized at the MP2/6-31G* level (Table S1). The MP2 models did not improve on the agreement with the parm94 charges over the HF/6-31G* models. For structure preparation in parm94 charge derivation, Hartree–Fock appeared to be near or at the limit of usefulness achievable by ab initio approaches.

**Figure 1:**
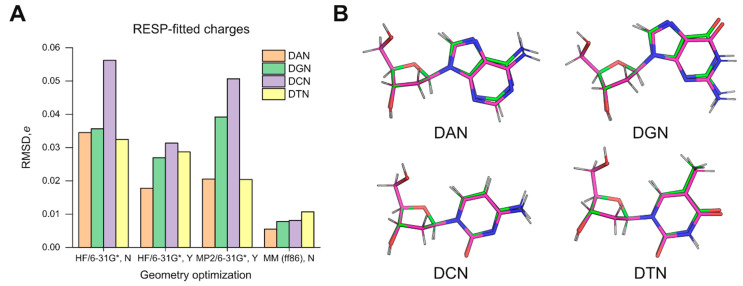
**Comparison of geometry optimization schemes for ff94 charge parameterization.** (**A**) Agreement with the parm94 charge set by RESP-fitted charges derived from structures optimized by ab initio (HF/6-31G* and MP2/6-31G*) and MM methods as described in the text. Y/N in the abscissa refers to whether the optimization was constrained (QM) or restrained (MM). Parametric values are provided in Table S1, Supplementary Materials. (**B**) Each pair of QM-optimized (magenta carbon) and MM-minimized (green carbon) structures were aligned by the five deoxyribose heavy ring atoms (C1’ to C4’ and O4’). The QM models were optimized with constraints targeted at the geometry specified for ff94 charge derivation. The MM minimization against the ff86 forcefield closely approached the geometric targets even without any strong restraints. See Table 1 for numerical values.

The results with HF/6-31G*-optimized structures as inputs for charge fitting prompted us to ask whether QM-based structures were necessarily better models for charge fitting over the MM-minimized structures (generated by the previous-generation ff86 forcefield) [18] used in the derivation of ff94. As the legacy AMBER4 program used to energy-minimize the structures for ff94 is no longer maintained in official AMBER repositories, we used AMBER5 (courtesy of Dr. Hector Baldoni, Universidad Nacional de San Luis) as the closest substitute. As inputs, we constructed deoxynucleosides based on the parameter set of ff86 (parm86). The resultant models were quite different in conformation and charge distribution from the specifications in Cieplak et al. [18]. Nevertheless, following energy minimization in the ff86 forcefield, the output structures were significantly closer in conformation than the HF/6-31G*-optimized models to the target geometries, even without the use of strong restraints to enforce agreement (Table S1). Subsequent charge fitting by ESP followed by multi-molecular RESP yielded atomic charges that agree better with parm94 by a factor of 3 (deoxythymidine) to over 6 (deoxycytidine) in RMSD over charges derived from HF/6-31G*-optimized structures (Figure 1A). The goodness-of-fit metrics were indistinguishable in all cases (Table S1), indicating that the differences in the charges did not originate with RESP fitting but were pre-existing in the structures.

To better understand the structural basis of the differences, we examined the optimized structures by HF/6-31G* and energy minimization in ff86. For each nucleoside, we aligned the pair of structures by the five atoms of the deoxyribose ring (C1’ to C4’ as well as O4’) (Figure 1B). In all cases, the RMSD values for these endocyclic atoms were below 0.01 Å. Since ff86-minimization closely achieved the Cremer–Pople pucker values specified by Cieplak et al. (Table 1) [18], the slight deviations in the pucker of the constrained QM structures were not the main contributor to the discrepancy in RESP-fitted charges. Thus, the overall geometries used in ff94 derivation appeared to fundamentally deviate from those predicted quanta mechanically. Since ESP fitting is known to be highly sensitive to conformation [15], one expects structural differences to be amplified and passed on to RESP, which uses the ESP results directly. As a result, the selection of MM-minimized models as structures for QM-based charge parameterization in the original derivation of ff94 would be a consequential choice, with implications for the parameterization of new residues for use with this forcefield. The adage: “All models are wrong, but some are useful,” famously attributed to the statistician George E.P. Box, appears to apply to this situation.

*A contemporary protocol for consistently parameterized DNA residues for ff94*. The better suitability of the AMBER-minimized models as input structures for QM-based charge fitting raises two potential challenges if they were to serve an accessible workflow for the community. The first is the general lack of availability of legacy versions of the AMBER software from the time that ff94 was developed. To overcome this, we verified that AMBER16 reproduced AMBER5 outputs to 10^−3^ or better when minimization is carried out in a flat dielectric corresponding to the gas phase (unit dielectric) (Table S2, Supplementary Materials).

The second challenge with generating appropriate MM-minimized models for ff94 concerns the input structures. Nucleoside structures are constructed in ff86 by a fragment-based approach that conjoins separately prepared bases and deoxyribose. The atomic charges were computed by ESP fitting from experimental structures of *N*9-methylpurine/*N*1-methylpyrimidine base and 1’-aminodeoxyribose analogs [13]. Sourcing appropriate experimental structures of such analogs would likely be generally problematic for novel entities. To overcome this, we asked whether the structures of the analogs could be adequately furnished by QM optimization of ab initio models. We therefore generated structures of various compounds in silico and compared their ESP-fitted atomic charges with reported values [13] computed from experimental structures. Charges in simple test compounds (water, formaldehyde, dimethyl ether, and methanol) were all well matched up to HF/6-31G** when the in silico structures were optimized at mp2/aug-cc-pVTZ (Table S3, Supplementary Materials). For nucleobases analogs (*N*9-methyladenine, *N*9-methylguanine, *N*1-methylcytosine, *N*1-methyladenine, and *N*1-methyluracil) and 1’-aminodeoxyribose, structures optimized at HF/6-311G++(3df,2p) level gave ESP-fitted charges at the HF/STO-3G level (which was used in deriving ff86) with comparable RMSDs (Table S4, Supplementary Materials). More computationally costly structures at a higher level of theory or larger basis set did not furnish significantly better matched charges. We therefore conclude that base fragments polished at the HF/6-311G++(3df,2p) level represented sufficiently accurate models for constructing nucleosides for the ff86 forcefield [21,22].

**Figure 2:**
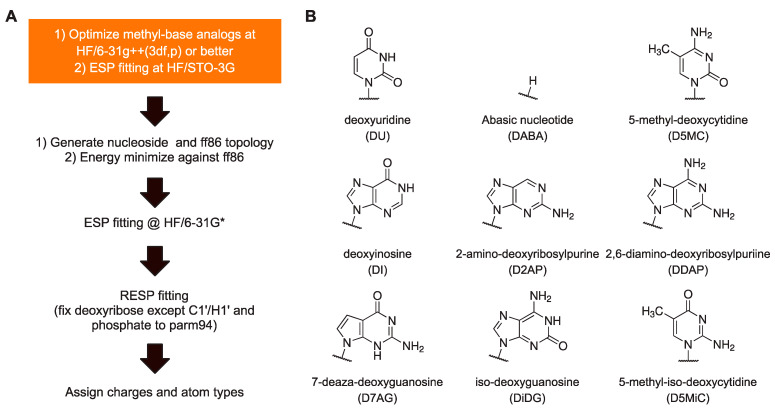
**Self-consistent parameterization of DNA residues for ff94 and derived (parmbsc1, and OL15) AMBER forcefields.** (**A**) Workflow of the procedure. The steps in the orange box are described in the main text. A detailed summary of generating the ff86 topology is provided in Supplemental Methods. The remaining steps are exactly as practiced elsewhere [18]. (**B**) Residues derived using this protocol. Final RESP-fitted charges are listed in Table S5 together with a comparison with previously reported values in the literature.

To summarize, our protocol for parameterizing new residues that retain maximum self-consistency with the AMBER charge set from parm94 up to the most current (parmbsc1 and OL15) is as follows (Figure 2A). Perform HF optimization of the methyl base analog at as large a basis set as practical, e.g., HF/6-31G++(3df,p), followed by ESP fitting at HF/STO-3G. Based on these ESP charges, generate an ff86-compatible structure using charges from parm86 for the deoxyribose. A detailed description of this procedure is provided in Supplemental Methods. Energy-minimize this structure in a flat gas-phase dielectric while applying restraints on H5’ and H3’ to remain in *trans* with bonded heavy atoms. Continue with ESP and RESP as usual [18]. We note that the steps prior to ESP are straightforward and computationally inexpensive relative to ab initio optimization of whole nucleosides at comparable levels of theory and size of basis set.

The limitations of our recipe are the same as those for the ff94-based forcefields. The parm94 charge set used only a single conformation of the deoxyribose (C2’-endo) and backbone torsions. For self-consistency with the canonical residues, we did not incorporate multiple conformations in the RESP fitting [20,23]. One could of course parameterize the canonical and new residues completely anew with multi-conformational fitting, but the resultant charges and simulations would not be consistent with those using the authentic parm94-derived parameter set. Finally, residues with radically novel structures may not be adequately supported by the existing set of bond and dihedral types in the ff86 forcefield and require additional parameterization regardless of the method of charge derivation.

Having established a protocol for setting the atomic charges of residues to maximize consistency with the existing residues in ff94, we set out to parameterize a panel of useful non-canonical DNA residues (Figure 2B). Since *N*1-methyluracil had been parameterized for uridine for ff86 [13], this recipe immediately yielded deoxyuridine. The recipe also yielded an abasic residue (hydrogen-capped C1’), another experimentally useful construct, by using CH_4_ as a methyl analog of a hydrogen atom. Other methyl analogs were drawn from residues that have previously been parameterized as well as commercially available residues: inosine, 2-aminopurine (2AP), 2,6-diaminopurine (DAP), 5-methylcytosine (5-MC), iso-guanine (iso-dG), and 5-methyl-iso-cytosine (5-methyl-iso-dC). All *N*-methyl-base analogs were optimized at the HF/6-311++g(3df,2p) level and conjoined with 1-aminodeoxyribose exactly as described for ff86 [22] (see Supplemental Methods). Roughly, the excess partial charges incurred from removing the methyl and amino substituents from the base and deoxyribose were absorbed into N1/N9 and C1’ atoms. All nucleosides generated were associated with the same set of charges for the sugar atoms for energy minimization. Following ESP fitting at the HF/STO-3G level, the new residues were RESP fitted globally with the four canonical DNA residues with the charges of the phosphate and deoxyribose (except for C1’and H1’) fixed to parm94 values. The chemical modifications in the presented non-canonical bases were assigned by analog using existing bond and dihedral types. The full set of RESP-fitted charges of the nine non-canonical bases, parameterized for parmbsc1 are given in Table S5, Supplementary Materials, with literature values [29,30,31,32,33,34] where available. Parenthetically, an inspection of Table S5 shows the RMS deviations between our charges and those from the literature to be on the same order of magnitude, and in the same direction, as the differences between charges derived from QM-based models for the canonical residues in parm94 (c.f., Figure 1A).

*Is global fitting essential for parameterizing new residues?* In principle, self-consistency is most retained if all residues are fitted simultaneously. The RESP implementation in AMBER provides this, and we fitted all nine non-canonical residues together with the four canonical residues. To render new residues transparent to the canonical bases, we fixed (as others have carried out) the deoxyribose and phosphate charges to the parm94 values. In global fitting, the optimization of each parameter set is not independent but subject to influence by the full data set. To evaluate the sensitivity of RESP-fitted charges to the statistics of global fitting, we compared the RESP charges of non-canonical residues that were fitted globally with charges derived from fitting the same residues individually with the canonical ff94 bases. Using inosine, 2-AP, and 5-MC as examples, we found negligible differences, no more than 10^−5^ in RMSD between the two approaches (Table S6, Supplementary Materials). This robustness appeared to reflect the greatly reduced number of statistical degrees of freedom when the phosphate and most of the deoxyribose charges were fixed to the parm94 values. The practical implication is that future residues could be conveniently added on their own, even one at a time, suggesting a good degree of “future-proofing” in the procedure.

**Figure 3:**
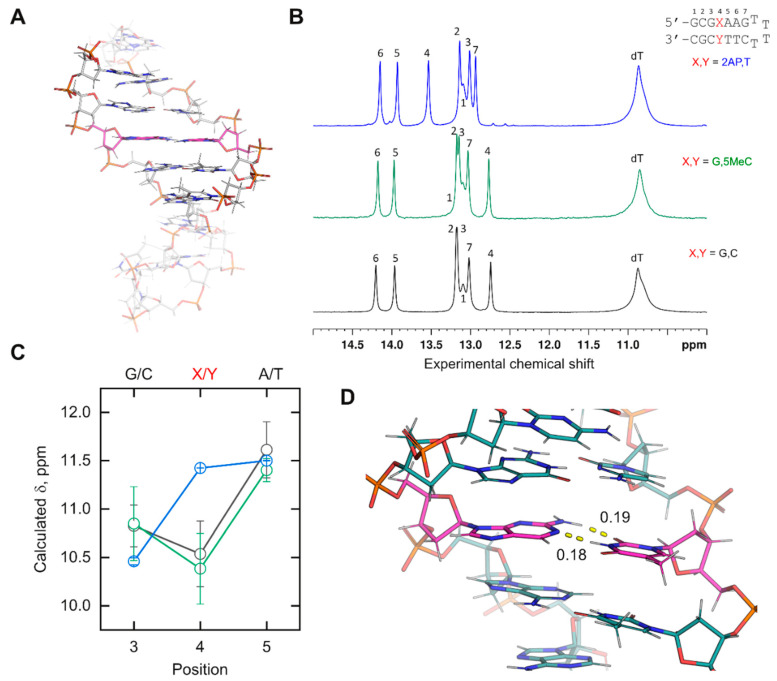
**Comparison of experimental ^1^H chemical shifts with predictions using simulated models of DNA duplexes harboring non-canonical residues.** (**A**) Hairpin cassette designed for this study. The entire hairpin was simulated as an unrestrained *NPT* ensemble, and an internal 5 bp stack, rendered in opaque colors, was used for chemical shift calculations. The central probe residue position in the duplex is indicated with magenta carbon. (**B**) Experimental imino ^1^H spectra of three test sequences, referenced against DSS and optimally resolved at 288 K. Peaks were assigned by ^1^H-^1^H NOESY experiments (not shown). (**C**) Calculated chemical shifts for test sequences, referenced against the averaged computed methyl ^1^H of an optimized DSS structure. Colors follow the spectra in Panel B. Points represent the means ± standard deviation of the triplicate averaged structures. (**D**) Illustrative averaged conformation of the 2AP:T base pair. Watson–Crick bonds are shown with units in nm.

*Demonstration of newly incorporated DNA residues*. The chemical shifts of imino protons are sensitive probes of nucleic acid conformation in solution. Chemical shifts depend on the nature of the nucleobase, the exchange behavior with bulk water for rapidly exchanging (broad) residues, and the local environment. We therefore tested several of the newly parameterized residues in Figure 2B by characterizing their experimental and computed chemical shift in a mixed-sequence duplex DNA construct. We designed a DNA hairpin in which a probe base **X** was part of an internal sequence 5’-d(CG**X**AA)-3’ (Figure 3A). To maximize the duplex structure of the cassette, the stack was flanked by a standard cassette consisting of an extra base pair from the terminus on one side and a T_4_ hairpin on the other. All bases in the cassette were canonical DNA. A reference hairpin harboring 5’-d(CG**G**AA)-3’ serves as a control.

Experimental ^1^H chemical shifts were measured by NMR spectroscopy in NaH_2_PO_4_/Na_2_HPO_4_ buffer (20 mM, pH 6.4) containing 10% D_2_O and 50 mM NaCl (Figure 3B). For MD simulations, the unrestrained *NPT* ensemble was sampled in explicit TIP3P water with nominally 50 mM Na^+^/Cl^−^ counterions at 298 K (see Supplementary Materials). Supported by the strongly convergent trajectory (over 200 ns) based on the RMSD of atomic coordinates, we generated averaged structures of the 5 bp stack from non-overlapping 50 ns segments of trajectory (Figure S1, Supplementary Materials). Given the substantial atom count of the stack, we optimized the averaged structures by energy minimization against parmbsc1 with a complement of TIP3P water and Na^+^/Cl^−^ ions at the same nominal concentration as in the NMR samples. For each minimized structure, chemical shifts were computed at the B3LYP/6-31G* level in implicit water. Following this procedure, we compared the imino proton chemical shifts for the inner three positions of the stack, i.e., d(G**X**A), with the experimental values. Chemical shifts for distal base pairs of the stack, which were fully exposed to solvent in the calculations, were expected to diverge strongly from the experimental values.

For the canonical-only control, the imino protons of the inner triplet from the simulation (Figure 3C) showed the same rank order as the experimental chemical shifts. A lack of quantitative agreement with the experimental values might be expected given the structure minimization (which was needed but also perturbative), forgoing the computational demands of larger basis sets, and limitations in capturing hydration effects with implicit water [35]. To test the consistency of the results, we examined a hairpin in which the probe dG was paired with 5-methyl-dC. The experimental imino ^1^H chemical shifts varied at all positions by ±0.03 ppm or less between the reference and methyl-substituted hairpin. We were encouraged to find that the computed imino proton shifts of the inner triplet overlapped closely within their standard deviations between the two simulated hairpins. This was not a trivial result, as a comparison of D5MC (Table S5) shows charge redistribution relative to DC (Table S4) over multiple atoms.

We next tested d(2AP) paired with dT, whose imino peak (contributed by T) was significantly downshifted, by 0.8 ppm, relative to the other two base pairs probes. We found that the computed probe chemical shift was also downshifted, inverting its position with position 3 as observed experimentally. While imino proton shifts from T are generally downfield from G counterparts, the order of the computed chemical shifts for the mixed purine/pyrimidine 5-d(G-2AP-A)-3′ triplet remained in agreement with the experimental one, lending further credence to the physical relevance of the simulated DNA models harboring novel residues. Examination of the trajectory showed that the 2AP paired with the T via expected Watson–Crick interactions (Figure 3D). Similar to active efforts for RNA [36,37], the semi-quantitative agreement with experimental chemical shifts suggests that ns-timescale simulations represent reasonable starting points for chemical shift prediction in the case of duplex DNA structures. Currently, classical forcefields admit a tradeoff in neglecting nuclear quantum effects [9,11] in their treatment of H-bonding. One may envision that, as the computational burden of AIMD becomes more tractable for macromolecules, prediction of the conformational and hydration contributions to experimental spectroscopic observables would be further improved.

## 4. Conclusions

Benchmarked against the parm94 charge set of canonical DNA residues, RESP-fitted charges derived from QM-optimized models exhibit RMS deviation on the order of 10%, while those from MM-minimized (ff86-based) structures deviate no more than 1%. The higher self-consistency in MM-minimized structures over QM models for charge derivation of DNA residues for current AMBER nucleic acids forcefields thus motivates the use of MM-minimized structures for the parameterization of new residues. To bridge the gaps left by the approach [18] and legacy software used in the development of the parm94 DNA charge set, which is used also in the most current updates (parmbsc1 and OL15), we have devised a workflow for generating appropriate structural models from ff86 using contemporary computational methodologies. We presented a panel of nine non-canonical residues, some of which have been previously derived using ab initio structural models. While we do not suggest that RESP-fitted charges of residues derived from QM-optimized structures are invalid for their intended purposes, they are demonstrably less self-consistent with the charge set of the original canonical residues and can be readily improved through a well-defined change in structure preparation (Figure 2A). As high-level optimization of whole nucleosides, particularly of large and complex bases (e.g., dye-conjugation or heavy metal substitution), remains computationally demanding, we suggest that the proposed protocol is compelling in providing higher-quality charges at reduced computational effort.

## Figures and Tables

**Table 1:** **QM- and MM-optimized geometries of canonical DNA nucleosides.** Reference values are quoted exactly as reported by Cieplak et al. [18]. Parametric values from QM (HF/6-31G*) and MM optimizations (against the ff86 forcefield) are given to one additional significant figure, with % deviation from the reference values in parenthesis. In all cases, H5’ and H3’ are fixed in *trans* with the bonded heavy atoms [18]. The constrained QM optimizations imposed additional dihedral constraints to satisfy the reference values in the initial structure. The corresponding geometry of 1-NH_2_-deoxyribose is provided to assess the impact of the base in the nucleosides.

	*q*, Å	*W*, °	γ, °	χ, °
**DAN**				
Reference	0.38	151.9	58.5	210.0
QM	0.337 (−11.3%)	163.62 (7.7%)	51.87 (−11.3%)	227.63 (8.4%)
QM constrained	0.379 (−0.3%)	152.56 (0.4%)	58.50	210.00
MM	0.380	151.84 (−0.04%)	60.40 (3.2%)	206.69 (−1.6%)
**DGN**				
Reference	0.38	151.4	58.5	209.9
QM	0.338 (−11.0%)	164.37 (8.6%)	50.91 (−13.0%)	231.60 (10.3%)
QM constrained	0.376 (−1.1%)	152.01 (0.4%)	58.50	209.90
MM	0.378 (−0.5%)	151.23 (−0.1%)	59.56 (1.8%)	208.74 (−0.5%)
**DCN**				
Reference	0.38	149.2	58.9	209.7
QM	0.337 (−11.3%)	159.50 (6.9%)	54.40 (−7.6%)	205.82 (−1.9%)
QM constrained	0.379 (−0.3%)	149.74 (0.4%)	58.90	209.70
MM	0.383 (0.8%)	149.46 (0.2%)	60.85 (4.0%)	210.27 (0.2%)
**DTN**				
Reference	0.38	149.1	58.4	215.7
QM	0.345 (−9.2%)	159.73 (7.1%)	52.23 (−10.6%)	226.54 (5.0%)
QM constrained	0.383 (0.8%)	149.59 (0.3%)	58.40	215.70
MM	0.382 (0.5%)	149.62 (0.3%)	60.49 (3.6%)	215.43 (−0.1%)
1-NH_2_-deoxyribose (QM)	0.332	163.03	51.54	

## Data Availability

The data presented in this study are available on reasonable request from the corresponding authors.

## Supplementary Materials

The following are available online at https://www.mdpi.com/article/10.3390/life12050666/s1, Supplemental Methods. Table S1. RESP-fitted atomic charges of QM- and MM-optimized canonical DNA nucleosides. Table S2. Reproducibility of legacy minimization results by contemporary versions of AMBER. Table S3. Comparison of fits to charge models between experimental and optimized ab initio structures. Table S4. QM-optimized structures of nucleobase fragment analogs reproduce ff86 atomic charges. Table S5. Final RESP-fitted atomic charges and comparison with literature. Table S6. Perturbation of global fitting on RESP-fitted charges. Figure S1. Equilibration of the DNA hairpin in unrestrained MD simulations.
