# Supplementary material for: Self-Consistent Parameterization of DNA Residues for the Non-Polarizable AMBER Force Fields
Source: Life (Basel). 2022 Apr 30;12(5):666. doi: 10.3390/life12050666 (PMC9143812; doi:10.3390/life12050666)
Supplement: Supplementary file 1 [file life-12-00666-s001.zip › life-1686107-supplementary.pdf]

life

MDPI

*Supplementary Materials*

# Self-Consistent Parameterization of DNA Residues for the Non-Polarizable AMBER Force Fields

**Amelia L. Schneider,[1] Amanda V. Albrecht,[1] Kenneth Huang,[1] Markus W. Germann,[1,2,*] and Gregory M. K. Poon[1,3,*]**

[1] Department of Chemistry, Georgia State University, Atlanta, GA 30303, USA; aschneider@gsu.edu (A.L.S.); aalbrecht1@gsu.edu (A.V.A.); khuang8@gsu.edu (K.H.)
[2] Department of Biology, Georgia State University, Atlanta, GA 30303, USA
[3] Center for Diagnostics and Therapeutics, Georgia State University, Atlanta, GA 30303, USA
* Correspondence: mwg@gsu.edu (M.W.G.); gpoon@gsu.edu (G.M.K.P.)

**INDEX TO THE SUPPLEMENT**

SUPPLEMENTAL METHODS
SUPPLEMENTAL TABLES AND FIGURES

## SUPPLEMENTAL METHODS

*Restrained electrostatic potential (RESP) fitting*. For the four canonical residues, ESPs were computed at HF/6-31G* with Gaussian 16 with the IOp keyword IOP(6/33=2). The outputs were processed by *espgen* to generate input files for RESP. Following the original workflow in ff94 [18], each nucleoside was combined with a dimethylphosphate (DMP) anion that had been HF/6-31G*-optimized to match the specified dihedrals of C1-O1-P-O2 = O1-P-O2-C2 = 73.1°. Two constraints were imposed: 1) the DMP moiety was assigned to carry all of the −1 charge, and 2) the methyl groups in DMP and terminal -OH groups of the nucleoside sum to zero. Across the nucleosides, the deoxyribose and backbone atoms were set to share charges, with exception to C1' and H1', which floated with each nucleobase. Hydrogen atoms in each methyl group were equivalenced. Fitting occurred in two stages. In the first, a hyperbolic restraint of 0.0005 A.U. was applied to all heavy atoms. In the second stage, a stronger restraint of 0.001 A.U. was used to refit methyl and methylene hydrogens only, while fixing all other atoms to their values from the first stage.

RESP charges for the non-canonical bases were globally fitted with the canonical bases. The charges of the canonical residues were constrained to their values in ff94 and imposed on the deoxyribose (except C1' and H1') and phosphate atoms across the entire set. The only floating charges were therefore the nucleobase atoms of the new bases. The same two-stage procedure was otherwise followed.

*Parameterization of ff86 nucleosides*. Following the fragment-based approach reported by Weiner et al [22], nucleosides were constructed by combining 1-aminodeoxyribose and a *N*9-methylpurine or *N*1-methylpyrimidine. For canonical residues, the atomic charges of the fragments were reported by Singh and Kollman [13]. Essentially, and unlike the procedure taken for amino acids, the excess atomic charges incurred from the removal of the amino and methyl substituents in the fragments were locally absorbed into the C1' and N1/N9 atoms. However, this was not done identically for each residue, but via the following rule, which was not explicitly described but inferred from Figure 2 in the Weiner et al paper [22]. For deoxycytidine only, the excess charges from both the amino and methyl groups (−0.237 + 0.055 = −0.182) were summed and assigned exclusively to C1' i.e., 0.558 – 0.182 = 0.376. The *N*1 atom in cytidine retained the charge in *N*1-methylcytosine (−0.187). The adjusted deoxyribose in deoxycytidine was then templated to the other nucleosides. Specifically, the glycosidic N atoms were adjusted by an amount equal to the

charge of the adjusted deoxyribose (0.055) plus the base of interest without the methyl group, with *no additional adjustment to the deoxyribose*:

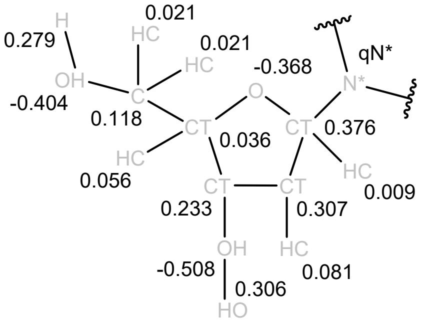


$$qN^{*} = qN^{*}_{ESP} - [0.055 + q(\text{base without } CH_3)]$$

Charge values in black are taken from Weiner et al [22]. These residual adjustments to the glycosidic N atoms in deoxythymidine, deoxyadenosine, and deoxyguanosine were −0.016, 0.010, and 0.020 respectively.

## INDEX TO SUPPLEMENTAL TABLES AND FIGURES

Table S1. RESP-fitted atomic charges of QM- and MM-optimized canonical DNA nucleosides
Table S2. Reproducibility of legacy minimization results by contemporary versions of AMBER
Table S3. Comparison of fits to charge models between experimental and optimized *ab initio* structures
Table S4. QM-optimized structures of nucleobase fragment analogs reproduce ff86 atomic charges
Table S5. Final RESP-fitted atomic charges and comparison with literature
Table S6. Perturbation of global fitting on RESP-fitted charges
Figure S1. Equilibration of the DNA hairpin in unrestrained MD simulations

**Table S1.** RESP-fitted atomic charges of QM- and MM-optimized canonical DNA nucleosides.

Reference values are atomic charges in ff94. The other charges are derived from QM- and MM-optimized models. Geometric specifications refer to the reference summary parameters in Table 1. Y/N refers to whether the optimization was constrained (QM) or restrained (MM). In all cases, H5' and H3' are constrained in the QM optimization and restrained in the MM minimization. Following geometry optimization, ESP fitting was performed at the HF/6-31G* level and the output passed onto the two-step RESP-fitting procedure for ff94 [18] described in *Supplemental Methods*. In particular, C1' and H1' from deoxyribose are not equivalenced and are reported with the bases. All parametric values are reported at the full precision available.

**Nucleobase + non-equivalenced C1'/H1'**

| DAN | | Subject to ($q$, $W$, $\gamma$, $\chi$) specifications | | | |
|---|---|---|---|---|---|
| | | N | Y | Y | N |
| Position | ff94 | HF/6-31G * | HF/6-31G | MP2/6-31G* | MM(ff86) |
| C1' | 0.0431 | 0.107555 | 0.070706 | 0.095582 | 0.032834 |
| H1' | 0.1838 | 0.157014 | 0.165702 | 0.165432 | 0.181715 |
| N9 | −0.0268 | −0.062163 | −0.050772 | −0.047366 | −0.027447 |
| C8 | 0.1607 | 0.144636 | 0.174481 | 0.144843 | 0.168181 |
| H8 | 0.1877 | 0.191428 | 0.185140 | 0.198879 | 0.183289 |
| N7 | −0.6175 | −0.590904 | −0.614204 | −0.604670 | −0.615574 |
| C5 | 0.0725 | 0.019601 | 0.055616 | 0.057751 | 0.074189 |
| C6 | 0.6897 | 0.696800 | 0.706390 | 0.705629 | 0.680556 |
| N6 | −0.9123 | −0.914322 | −0.937631 | −0.942502 | −0.906295 |
| H61 | 0.4167 | 0.413237 | 0.419008 | 0.421292 | 0.414963 |
| H62 | 0.4167 | 0.413237 | 0.419008 | 0.421292 | 0.414963 |
| N1 | −0.7624 | −0.772924 | −0.767573 | −0.756753 | −0.757845 |
| C2 | 0.5716 | 0.606425 | 0.578445 | 0.544437 | 0.573199 |
| H2 | 0.0598 | 0.048195 | 0.055215 | 0.069074 | 0.057312 |
| N3 | −0.7417 | −0.775456 | −0.752867 | −0.734434 | −0.734724 |
| C4 | 0.3800 | 0.460465 | 0.421950 | 0.399293 | 0.370340 |
| RMSD | | 0.034506 | 0.017795 | 0.020558 | 0.005530 |

| DGN | | Subject to ($q$, $W$, $\gamma$, $\chi$) specifications | | | |
|---|---|---|---|---|---|
| | | N | Y | Y | N |
| Position | ff94 | HF/6-31G * | HF/6-31G | MP2/6-31G* | MM(ff86) |
| C1' | 0.0358 | 0.114539 | 0.091560 | 0.133338 | 0.015220 |
| H1' | 0.1746 | 0.130511 | 0.134362 | 0.128628 | 0.173741 |
| N9 | 0.0577 | 0.033421 | 0.023835 | −0.001260 | 0.075520 |
| C8 | 0.0736 | 0.068252 | 0.094789 | 0.086033 | 0.070204 |
| H8 | 0.1997 | 0.202180 | 0.196165 | 0.204985 | 0.200477 |
| N7 | −0.5725 | −0.552034 | −0.574758 | −0.568379 | −0.572997 |
| C5 | 0.1991 | 0.147480 | 0.200138 | 0.206863 | 0.201942 |
| C6 | 0.4918 | 0.545239 | 0.501894 | 0.494264 | 0.489296 |
| O6 | −0.5699 | −0.552780 | −0.546155 | −0.546107 | −0.570949 |
| N1 | −0.5053 | −0.567332 | −0.521219 | −0.533157 | −0.496718 |
| H1 | 0.3520 | 0.363692 | 0.354408 | 0.359601 | 0.350301 |
| C2 | 0.7432 | 0.762123 | 0.709546 | 0.708897 | 0.739729 |
| N2 | −0.9230 | −0.924639 | −0.898491 | −0.877218 | −0.922955 |

| | | | | | |
|---|---|---|---|---|---|
| H21 | 0.4235 | 0.401670 | 0.395934 | 0.383862 | 0.422815 |
| H22 | 0.4235 | 0.401670 | 0.395934 | 0.383862 | 0.422815 |
| N3 | −0.6636 | −0.635772 | −0.622291 | −0.609413 | −0.657950 |
| C4 | 0.1814 | 0.204604 | 0.192961 | 0.182979 | 0.169166 |
| RMSD | | 0.035655 | 0.026965 | 0.039200 | 0.007824 |

| **DCN** | | **Subject to (*q*, *W*, γ, χ) specifications** | | | |
|---|---|---|---|---|---|
| | | N | Y | Y | N |
| Position | ff94 | HF/6-31G * | HF/6-31G | MP2/6-31G* | MM(ff86) |
| C1' | −0.0116 | 0.131453 | 0.040351 | 0.069357 | −0.019945 |
| H1' | 0.1963 | 0.122635 | 0.173546 | 0.167499 | 0.200608 |
| N1 | −0.0339 | −0.075401 | −0.065204 | −0.072971 | −0.049423 |
| C6 | −0.0183 | −0.034262 | −0.015413 | 0.005525 | −0.019243 |
| H6 | 0.2293 | 0.213119 | 0.219090 | 0.218902 | 0.223781 |
| C5 | −0.5222 | −0.474449 | −0.500410 | −0.495985 | −0.509247 |
| H5 | 0.1863 | 0.182040 | 0.188226 | 0.190456 | 0.184294 |
| C4 | 0.8439 | 0.766984 | 0.786811 | 0.745479 | 0.844565 |
| N4 | −0.9773 | −0.908439 | −0.925120 | −0.879898 | −0.981574 |
| H41 | 0.4314 | 0.398940 | 0.404086 | 0.384929 | 0.431705 |
| H42 | 0.4314 | 0.398940 | 0.404086 | 0.384929 | 0.431705 |
| N3 | −0.7748 | −0.760225 | −0.764340 | −0.731506 | −0.781861 |
| C2 | 0.7959 | 0.821355 | 0.832750 | 0.790352 | 0.813377 |
| O2 | −0.6548 | −0.639867 | −0.649846 | −0.639288 | −0.659086 |
| RMSD | | 0.056237 | 0.031343 | 0.050659 | 0.008128 |

| **DTN** | | **Subject to (*q*, *W*, γ, χ) specifications** | | | |
|---|---|---|---|---|---|
| | | N | Y | Y | N |
| Position | ff94 | HF/6-31G * | HF/6-31G | MP2/6-31G* | MM(ff86) |
| C1' | 0.068 | 0.110128 | 0.077476 | 0.092102 | 0.055576 |
| H1' | 0.1804 | 0.160001 | 0.172244 | 0.173644 | 0.182462 |
| N1 | −0.0239 | −0.025585 | −0.017366 | −0.017414 | −0.030756 |
| C6 | −0.2209 | −0.280735 | −0.267025 | −0.253458 | −0.207161 |
| H6 | 0.2607 | 0.282872 | 0.272476 | 0.284520 | 0.254509 |
| C5 | 0.0025 | 0.002703 | 0.001983 | 0.008076 | −0.000128 |
| C7 | −0.2269 | −0.241687 | −0.250221 | −0.268888 | −0.258242 |
| H71 | 0.077 | 0.080609 | 0.083468 | 0.087809 | 0.085186 |
| H72 | 0.077 | 0.080609 | 0.083468 | 0.087809 | 0.085186 |
| H73 | 0.077 | 0.080609 | 0.083468 | 0.087809 | 0.085186 |
| C4 | 0.5194 | 0.598880 | 0.596151 | 0.557955 | 0.530958 |
| O4 | −0.5563 | −0.562337 | −0.561573 | −0.557381 | −0.560811 |
| N3 | −0.434 | −0.488988 | −0.489953 | −0.460458 | −0.438076 |
| H3 | 0.342 | 0.340992 | 0.341802 | 0.336661 | 0.342964 |
| C2 | 0.5677 | 0.598081 | 0.598190 | 0.565766 | 0.573119 |
| O2 | −0.5881 | −0.593328 | −0.595976 | −0.586773 | −0.590316 |
| RMSD | | 0.032454 | 0.028696 | 0.020406 | 0.010691 |

**Deoxyribose (except C1'/H1') and phosphate**

| | | Subject to ($q$, $W$, $\gamma$, $\chi$) specifications | | | |
|---|---|---|---|---|---|
| | | N | Y | Y | N |
| Position | ff94 | HF/6-31G * | HF/6-31G | MP2/6-31G* | MM(ff86) |
| H5T | 0.4422 | 0.446230 | 0.445388 | 0.435760 | 0.439483 |
| O5' | −0.6318 | −0.636987 | −0.633390 | −0.623394 | −0.625961 |
| C5' | −0.0069 | 0.004901 | −0.000928 | −0.009135 | −0.000599 |
| H5'1 | 0.0754 | 0.072676 | 0.071630 | 0.076062 | 0.072956 |
| H5'2 | 0.0754 | 0.072676 | 0.071630 | 0.076062 | 0.072956 |
| C4' | 0.1629 | 0.167846 | 0.204110 | 0.210710 | 0.161097 |
| H4' | 0.1176 | 0.106575 | 0.102110 | 0.107438 | 0.118168 |
| O4' | −0.3691 | −0.389816 | −0.396500 | −0.407692 | −0.363252 |
| C3' | 0.0713 | 0.112604 | 0.072212 | 0.035804 | 0.076272 |
| H3' | 0.0985 | 0.079566 | 0.089587 | 0.099671 | 0.094382 |
| C2' | −0.0854 | −0.061643 | −0.067813 | −0.065441 | −0.087246 |
| H2'1 | 0.0718 | 0.053208 | 0.065339 | 0.069370 | 0.074064 |
| H2'2 | 0.0718 | 0.053208 | 0.065339 | 0.069370 | 0.074064 |
| O3' | −0.6549 | −0.663996 | −0.659344 | −0.643722 | −0.655119 |
| H3T | 0.4396 | 0.440127 | 0.442018 | 0.431357 | 0.439079 |
| | | | | | |
| P | 1.1659 | 1.222739 | 1.222542 | 1.222350 | 1.222350 |
| OP1 | −0.7761 | −0.791043 | −0.791334 | −0.791383 | −0.791383 |
| OP2 | −0.7761 | −0.791043 | −0.791334 | −0.791383 | −0.791383 |
| O5' | −0.4954 | −0.519575 | −0.516493 | −0.514824 | −0.514824 |
| O3' | −0.5232 | −0.535703 | −0.528709 | −0.527277 | −0.527277 |
| RMSD | | 0.020729 | 0.019251 | 0.022258 | 0.014550 |

**Goodness-of-fit metrics**[a]

| | Step 1 | | | | Step 2 | | | |
|---|---|---|---|---|---|---|---|---|
| | ISS | RSS | SE | RRMS | ISS | RSS | SE | RRMS |
| HF/6-31G*, N | 17.656 | 0.021 | 0.00528 | 0.03466 | 17.656 | 0.022 | 0.00535 | 0.03510 |
| HF/6-31G*, Y | 17.637 | 0.021 | 0.00525 | 0.03447 | 17.637 | 0.021 | 0.00530 | 0.03478 |
| MP2/6-31G*, Y | 17.715 | 0.021 | 0.00527 | 0.03458 | 17.715 | 0.022 | 0.00534 | 0.03505 |
| MM (ff86) | 18.011 | 0.021 | 0.00524 | 0.03404 | 18.011 | 0.022 | 0.00533 | 0.03465 |

[a] ISS, initial sum of squares; RSS, residual sum of squares; $\mathrm{SE}=\sqrt{\mathrm{RSS}/\mathrm{N}}$, standard error of the estimates; $\mathrm{RRMS}=\sqrt{\mathrm{RSS}/\mathrm{ISS}}$, relative RMS due to ESP.

**Table S2.** Reproducibility of legacy minimization results by contemporary versions of AMBER.

Energy minimization by steepest descent in uniform gas phase using AMBER5 yielded the closest agreement with the reported geometries in ff94 derivation over other settings. The results in AMBER16 were obtained by turning off the Generalized Born implicit solvent (*igb=0*). In all cases, H5' and H3' were restrained in *trans* with bonded heavy atoms. minimizations were converged to an RMS value of $10^{-4}$ kcal $mol^{-1}$ $Å^{-1}$ for the energy gradient. All parametric values are reported at the full (single) precision reported by the software.

**Nucleobase + non-equivalenced C1'/H1'**

| DAN | parm94 | AMBER5 | AMBER16 |
|---|---|---|---|
| C1' | 0.0431 | 0.033003 | 0.032834 |
| H1' | 0.1838 | 0.181649 | 0.181715 |
| N9 | −0.0268 | −0.027489 | −0.027447 |
| C8 | 0.1607 | 0.168151 | 0.168181 |
| H8 | 0.1877 | 0.183301 | 0.183289 |
| N7 | −0.6175 | −0.615563 | −0.615574 |
| C5 | 0.0725 | 0.074204 | 0.074189 |
| C6 | 0.6897 | 0.680531 | 0.680556 |
| N6 | −0.9123 | −0.906281 | −0.906295 |
| H61 | 0.4167 | 0.414959 | 0.414963 |
| H62 | 0.4167 | 0.414959 | 0.414963 |
| N1 | −0.7624 | −0.757827 | −0.757845 |
| C2 | 0.5716 | 0.573170 | 0.573199 |
| H2 | 0.0598 | 0.057316 | 0.057312 |
| N3 | −0.7417 | −0.734703 | −0.734724 |
| C4 | 0.3800 | 0.370344 | 0.370340 |
| RMSD | | 0.005515 | 0.005530 |

| DGN | parm94 | AMBER5 | AMBER16 |
|---|---|---|---|
| C1' | 0.0358 | 0.015110 | 0.015220 |
| H1' | 0.1746 | 0.173741 | 0.173741 |
| N9 | 0.0577 | 0.075474 | 0.075520 |
| C8 | 0.0736 | 0.070274 | 0.070204 |
| H8 | 0.1997 | 0.200652 | 0.200477 |
| N7 | −0.5725 | −0.573131 | −0.572997 |
| C5 | 0.1991 | 0.201781 | 0.201942 |
| C6 | 0.4918 | 0.489288 | 0.489296 |
| O6 | −0.5699 | −0.570944 | −0.570949 |
| N1 | −0.5053 | −0.496754 | −0.496718 |
| H1 | 0.3520 | 0.350324 | 0.350301 |
| C2 | 0.7432 | 0.740021 | 0.739729 |
| N2 | −0.9230 | −0.923258 | −0.922955 |
| H21 | 0.4235 | 0.422893 | 0.422815 |
| H22 | 0.4235 | 0.422893 | 0.422815 |
| N3 | −0.6636 | −0.657994 | −0.657950 |
| C4 | 0.1814 | 0.169354 | 0.169166 |
| RMSD | | 0.007803 | 0.007824 |

| DCN | parm94 | AMBER5 | AMBER16 |
|---|---|---|---|

| | | | |
|---|---|---|---|
| C1' | −0.0116 | −0.020074 | −0.019945 |
| H1' | 0.1963 | 0.200679 | 0.200608 |
| N1 | −0.0339 | −0.049126 | −0.049423 |
| C6 | −0.0183 | −0.019435 | −0.019243 |
| H6 | 0.2293 | 0.223869 | 0.223781 |
| C5 | −0.5222 | −0.509065 | −0.509247 |
| H5 | 0.1863 | 0.184290 | 0.184294 |
| C4 | 0.8439 | 0.844480 | 0.844565 |
| N4 | −0.9773 | −0.981543 | −0.981574 |
| H41 | 0.4314 | 0.431685 | 0.431705 |
| H42 | 0.4314 | 0.431685 | 0.431705 |
| N3 | −0.7748 | −0.781912 | −0.781861 |
| C2 | 0.7959 | 0.813423 | 0.813377 |
| O2 | −0.6548 | −0.659234 | −0.659086 |
| RMSD | | 0.008133 | 0.008128 |

| DTN | parm94 | AMBER5 | AMBER16 |
|---|---|---|---|
| C1' | 0.0680 | 0.055726 | 0.055576 |
| H1' | 0.1804 | 0.182407 | 0.182462 |
| N1 | −0.0239 | −0.030772 | −0.030756 |
| C6 | −0.2209 | −0.207199 | −0.207161 |
| H6 | 0.2607 | 0.254528 | 0.254509 |
| C5 | 0.0025 | −0.000117 | −0.000128 |
| C7 | −0.2269 | −0.258219 | −0.258242 |
| H71 | 0.0770 | 0.085179 | 0.085186 |
| H72 | 0.0770 | 0.085179 | 0.085186 |
| H73 | 0.0770 | 0.085179 | 0.085186 |
| C4 | 0.5194 | 0.530955 | 0.530958 |
| O4 | −0.5563 | −0.560812 | −0.560811 |
| N3 | −0.4340 | −0.438061 | −0.438076 |
| H3 | 0.3420 | 0.342959 | 0.342964 |
| C2 | 0.5677 | 0.573099 | 0.573119 |
| O2 | −0.5881 | −0.590308 | −0.590316 |
| RMSD | | 0.010670 | 0.010691 |

**Deoxyribose (except C1'/H1') and phosphate**

| | parm94 | AMBER5 | AMBER16 |
|---|---|---|---|
| H5T | 0.4422 | 0.439492 | 0.439484 |
| O5' | −0.6318 | −0.625980 | −0.625961 |
| C5' | −0.0069 | −0.000596 | −0.000600 |
| H5'1 | 0.0754 | 0.072962 | 0.072956 |
| H5'2 | 0.0754 | 0.072962 | 0.072956 |
| C4' | 0.1629 | 0.160995 | 0.161094 |
| H4' | 0.1176 | 0.118194 | 0.118169 |
| O4' | −0.3691 | −0.363238 | −0.363251 |
| C3' | 0.0713 | 0.076323 | 0.076272 |
| H3' | 0.0985 | 0.094381 | 0.094382 |

| | | | |
|---|---|---|---|
| C2' | −0.0854 | −0.087307 | −0.087246 |
| H2'1 | 0.0718 | 0.074065 | 0.074064 |
| H2'2 | 0.0718 | 0.074065 | 0.074064 |
| O3' | −0.6549 | −0.655127 | −0.655119 |
| H3T | 0.4396 | 0.439085 | 0.439079 |
| | | | |
| P | 1.1659 | 1.222352 | 1.222350 |
| OP1 | −0.7761 | −0.791383 | −0.791383 |
| OP2 | −0.7761 | −0.791383 | −0.791383 |
| O5' | −0.4954 | −0.514836 | −0.514824 |
| O3' | −0.5232 | −0.527279 | −0.527277 |
| RMSD | | 0.014553 | 0.014550 |

**Table S3.** Comparison of fits to charge models between experimental and optimized *ab initio* structures.

Properties for each substance were computed following ESP fits with the MK scheme at the indicated theory and basis set. Parametric values in the shaded boxes are as reported by Singh and Kollman [13], using experimentally derived structures as referenced in their paper. Unshaded values were computed in this work using *ab initio* structures (in Z-matrix format) that have been geometry-optimized at the mp2/aug-cc-pVTZ level. The precision in the values is exactly as reported by Singh and Kollman or Gaussian. RRMS is the relative fit of the atomic charges to the ESP. RMSD refers to the differences between the shaded and unshaded values over all the atoms in each structure.

| | | **Water** HF/6-31G** | | | **Formaldehyde** HF/6-31G** | | | **Methanol** HF/6-31G** | | | **Dimethyl ether** HF/6-31G* | |
|---|---|---|---|---|---|---|---|---|---|---|---|---|
| | | 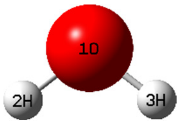 | | | 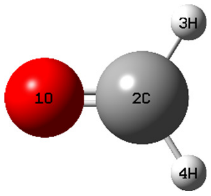 | | | 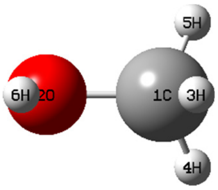 | | | 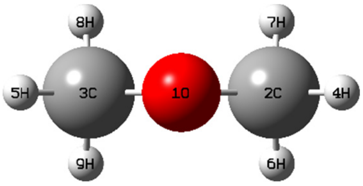 | |
| Point group | | $C_{2v}$ | | | $C_{2v}$ | | | $C_s$ | | | $C_{2v}$ | |
| Dipole, D | | 2.242 | 2.2365 | | 2.79 | 2.7992 | | 2.014 | 2.0173 | | 1.7968 | 1.6395 |
| Charge | | | | | | | | | | 1O | −0.4038 | −0.373840 |
| | | | | | | | | | | 2C | 0.0095 | −0.004309 |
| | | | | | | | 1C | 0.149 | 0.187178 | 3C | 0.0095 | −0.004309 |
| | 1O | −0.794 | −0.793710 | 1O | −0.463 | −0.455295 | 2O | −0.656 | −0.655165 | 4H | 0.0799 | 0.077640 |
| | 2H | 0.397 | 0.396855 | 2C | 0.421 | 0.402472 | 3H | −0.001 | −0.001455 | 5H | 0.0799 | 0.077640 |
| | 3H | 0.397 | 0.396855 | 3H | 0.021 | 0.026411 | 4H | 0.042 | 0.028724 | 6H | 0.0562 | 0.057967 |
| | | | | 4H | | | 5H | 0.042 | 0.028724 | 7H | 0.0562 | 0.055621 |
| | | | | | | | 6H | 0.424 | 0.411994 | 8H | 0.0562 | 0.055621 |
| | | | | | | | | | | 9H | 0.0562 | 0.057967 |
| RRMS | | 0.105 | 0.10903 | | 0.065 | 0.06466 | | 0.134 | 0.13092 | | 0.128 | 0.15227 |
| RMSD | | 0.000 | | | 0.011 | | | 0.018 | | | 0.012 | |

**Table S4.** QM-optimized structures of nucleobase fragment analogs reproduce ff86 atomic charges.

*Ab initio* models of 9-methyl-R (R = adenine, guanine), 1-methyl-Y (Y = cytosine, thymine), and 1-aminodeoxyribose (ADR) were geometry-optimized at the HF/6-311++G(3df,2p), MP2/6-311++G(3df,2p) (purines and ADR) or MP2/aug-cc-pVTZ (pyrimidines) in Z-matrices. Constraints were imposed on the nucleobase structures to enforce symmetry in the $C_s$ point group. Atom-centered charges were then computed by ESP fitting to the MK scheme at the HF/STO-3G level. Values reported by Singh and Kollman [13] (labeled REF) were used in deriving the atomic charges of nucleosides in the ff86 forcefield [22]. The atoms names and the precision in the reference charges follow the original literature.

**9-methylpurines**

| **9-CH₃-A** | | | | **9-CH₃-G** | | | |
|---|---|---|---|---|---|---|---|
| **Atom** | **REF** | **HF** | **MP2** | **Atom** | **REF** | HF | MP2 |
| N1 | −0.774 | −0.744875 | −0.728376 | N1 | −0.729 | −0.756373 | −0.729675 |
| C2 | 0.661 | 0.608821 | 0.579707 | C2 | 0.871 | 0.857102 | 0.843982 |
| N3 | −0.728 | −0.701292 | −0.678701 | N3 | −0.709 | −0.716057 | −0.697809 |
| C4 | 0.546 | 0.534725 | 0.503614 | C4 | 0.391 | 0.428305 | 0.400295 |
| C5 | −0.097 | −0.008791 | 0.004678 | C5 | −0.060 | −0.116132 | −0.092204 |
| C6 | 0.769 | 0.728420 | 0.710658 | C6 | 0.690 | 0.743430 | 0.696486 |
| N7 | −0.543 | −0.572461 | −0.560941 | N7 | −0.543 | −0.524076 | −0.509725 |
| C8 | 0.263 | 0.299245 | 0.269547 | C8 | 0.266 | 0.232672 | 0.199470 |
| N9 | −0.063 | −0.091396 | −0.070974 | N9 | −0.022 | 0.024654 | 0.035340 |
| N6 | −0.768 | −0.784211 | −0.775230 | O6 | −0.458 | −0.466985 | −0.450154 |
| H2 | −0.032 | −0.022335 | −0.014404 | N2 | −0.778 | −0.773501 | −0.766500 |
| H8 | 0.062 | 0.052360 | 0.059546 | H1 | 0.336 | 0.345623 | 0.334649 |
| C9M | −0.431 | −0.473507 | −0.475827 | H8 | 0.046 | 0.049594 | 0.058134 |
| HN6A | 0.335 | 0.323998 | 0.322231 | HN2A | 0.339 | 0.328853 | 0.328171 |
| HN68 | 0.324 | 0.338501 | 0.335330 | HN2B | 0.325 | 0.328276 | 0.325670 |
| HC9A | 0.158 | 0.167293 | 0.169263 | C9M | −0.459 | −0.477475 | −0.461568 |
| HC9B | 0.159 | 0.172753 | 0.174939 | HC9A | 0.163 | 0.160717 | 0.158149 |
| HC9C | 0.159 | 0.172753 | 0.174939 | HC9B | 0.164 | 0.165686 | 0.163645 |
| | | | | HC9C | 0.164 | 0.165686 | 0.163645 |
| Σ | 0.000 | 0.000001 | −0.000001 | Σ | −0.003 | −0.000001 | 0.000001 |
| RMSD | | 0.033232 | 0.041090 | RMSD | | 0.025861 | 0.024423 |

**1-methylpyrimidines**

| **1-CH₃-C** | | | | **1-CH₃-T** | | | |
|---|---|---|---|---|---|---|---|
| **Atom** | **REF** | **HF** | **MP2** | **Atom** | **REF** | HF | MP2 |
| N1 | −0.187 | −0.184349 | −0.171619 | N1 | −0.233 | −0.180511 | −0.160248 |
| C2 | 0.859 | 0.917796 | 0.885081 | C1 | 0.849 | 0.849187 | 0.811385 |
| N3 | −0.86 | −0.873709 | −0.865841 | N3 | −0.851 | −0.866361 | −0.832776 |
| C4 | 0.935 | 0.927792 | 0.933771 | C4 | 0.809 | 0.834598 | 0.792023 |
| C5 | −0.576 | −0.567869 | −0.571429 | C5 | −0.176 | −0.164261 | −0.149743 |
| C6 | 0.185 | 0.170583 | 0.172870 | C6 | 0.034 | 0.005720 | 0.008643 |
| O2 | −0.508 | −0.515315 | −0.505440 | O2 | −0.488 | −0.496127 | −0.480788 |
| N4 | −0.834 | −0.852182 | −0.853372 | O4 | −0.464 | −0.474602 | −0.460371 |
| C1M | −0.289 | −0.354829 | −0.369067 | C5M | −0.382 | −0.392113 | −0.402636 |

| | | | | | | | |
|---|---|---|---|---|---|---|---|
| HN4A | 0.153 | 0.153567 | 0.152251 | C1M | −0.251 | −0.329030 | −0.340033 |
| HN4B | 0.098 | 0.095509 | 0.098405 | H3 | 0.355 | 0.356185 | 0.344152 |
| H5 | 0.351 | 0.350757 | 0.350260 | H6 | 0.134 | 0.125927 | 0.126969 |
| H6 | 0.329 | 0.333776 | 0.333317 | HC5A | 0.119 | 0.116421 | 0.117727 |
| HC1A | 0.116 | 0.131746 | 0.136225 | HC5B | 0.111 | 0.116394 | 0.117658 |
| HC1B | 0.115 | 0.134980 | 0.138363 | HC5C | 0.111 | 0.116394 | 0.117658 |
| HC1C | 0.115 | 0.131746 | 0.136225 | HC1A | 0.118 | 0.131571 | 0.134371 |
| | | | | HC1B | 0.103 | 0.125304 | 0.128004 |
| | | | | HC1C | 0.103 | 0.125304 | 0.128004 |
| Σ | 0.002 | −0.000001 | 0.000000 | Σ | 0.001 | 0.000000 | −0.000001 |
| RMSD | | 0.024562 | 0.024159 | RMSD | | 0.026102 | 0.032364 |

**1-aminodeoxyribose**

| Atom | REF | HF | MP2 |
|---|---|---|---|
| O4 | −0.368 | −0.398285 | −0.407510 |
| C1' | 0.558 | 0.645792 | 0.636814 |
| C4' | 0.036 | 0.033739 | 0.076483 |
| C2' | −0.307 | −0.319766 | −0.299220 |
| C3' | 0.233 | 0.324864 | 0.270350 |
| N | −0.869 | −0.960932 | −0.944870 |
| O3' | −0.508 | −0.551424 | −0.522766 |
| C5' | 0.118 | 0.238037 | 0.200146 |
| H1' | 0.009 | −0.017274 | −0.015508 |
| HHA | 0.305 | 0.330877 | 0.326358 |
| HHB | 0.329 | 0.355927 | 0.349148 |
| H2' | 0.081 | 0.080602 | 0.077524 |
| H2' | 0.081 | 0.085355 | 0.082789 |
| H3' | 0.025 | 0.004437 | 0.015281 |
| H4' | 0.056 | 0.038744 | 0.034161 |
| H03' | 0.306 | 0.319956 | 0.304066 |
| O5' | −0.404 | −0.507202 | −0.473610 |
| H5' | 0.003 | −0.022468 | −0.017577 |
| H5' | 0.039 | 0.007728 | 0.014764 |
| H05' | 0.279 | 0.311293 | 0.293177 |
| Σ | 0.002 | 0.000000 | 0.000000 |
| RMSD | | 0.053999 | 0.039812 |

**Table S5.** Final RESP-fitted atomic charges and comparison with literature.

Charges given for the atoms that float (nucleobase and C1'/H1') in the RESP fit. The shared charges (remainder of the deoxyribose and phosphate) take on parm94 values such as those shown in Tables S1 and S2. Values are shown at the full precision computed or as reported in the literature. RMSD values relative to the charges reported in this work are reported at the precision of the literature values.

**Deoxyuridylate (DU)**

| Atom | This work |
|---|---|
| C1' | 0.070684 |
| H1' | 0.174261 |
| N1 | 0.019695 |
| C6 | −0.125917 |
| H6 | 0.246475 |
| C5 | −0.357841 |
| H5 | 0.176562 |
| C4 | 0.600792 |
| O4 | −0.575617 |
| N3 | −0.376442 |
| H3 | 0.319106 |
| C2 | 0.534284 |
| O2 | −0.584444 |

**Abasic nucleotide (DABA)**

| Atom | This work |
|---|---|
| C1' | −0.147704 |
| H1'1 | 0.134652 |
| H1'2 | 0.134652 |

**5-methyl-deoxycytidylate (D5MC)**

| Atom | This work | Lankas et al [34] | Carvalho et al [29] | Marco et al [32] | Schneider et al [31] |
|---|---|---|---|---|---|
| C1' | −0.008195 | −0.011600 | −0.0116 | −0.0116 | 0.0706 |
| H1' | 0.205216 | 0.196300 | 0.1963 | 0.1963 | 0.1591 |
| N1 | −0.062430 | −0.088800 | −0.0389 | −0.0339 | −0.1389 |
| C6 | −0.160421 | −0.082693 | −0.1485 | −0.0183 | −0.1438 |
| H6 | 0.261137 | 0.231353 | 0.2729 | 0.2293 | 0.2445 |
| C5 | −0.069175 | −0.157695 | −0.1927 | −0.3185 | −0.1052 |
| C7 | −0.244302 | −0.310870 | −0.2779 | −0.3646 | −0.2507 |
| H71 | 0.075076 | 0.097824 | 0.0943 | 0.1157 | 0.0793 |
| H72 | 0.075076 | 0.097824 | 0.0943 | 0.1157 | 0.0793 |
| H73 | 0.075076 | 0.097824 | 0.0943 | 0.1157 | 0.0793 |
| C4 | 0.617608 | 0.806980 | 0.7994 | 0.8439 | 0.7235 |
| N4 | −0.843162 | −0.976814 | −0.9571 | −0.9773 | −0.9037 |
| H41 | 0.397341 | 0.428276 | 0.4163 | 0.4314 | 0.3978 |
| H42 | 0.397341 | 0.428276 | 0.4163 | 0.4314 | 0.3978 |

| | | | | | |
|---|---|---|---|---|---|
| N3 | −0.723926 | −0.794899 | −0.7750 | −0.7748 | −0.7759 |
| C2 | 0.785741 | 0.827845 | 0.7401 | 0.7959 | 0.8559 |
| O2 | −0.656402 | −0.667531 | −0.6009 | −0.6548 | −0.6507 |
| RMSD | | 0.074840 | 0.0697 | 0.1089 | 0.0477 |

**Deoxyinosinate (DI)**

| Atom | This work | Lankas et al [34] | Marco et al [32] |
|---|---|---|---|
| C1' | 0.083144 | 0.043100 | 0.0431 |
| H1' | 0.157553 | 0.183800 | 0.1838 |
| N9 | −0.009820 | −0.009401 | −0.0138 |
| C8 | 0.109175 | 0.077630 | 0.0783 |
| H8 | 0.205646 | 0.211563 | 0.1773 |
| N7 | −0.577677 | −0.538290 | −0.5367 |
| C5 | 0.209141 | 0.049936 | 0.1663 |
| C6 | 0.522966 | 0.586826 | 0.5066 |
| O6 | −0.573726 | −0.581501 | −0.5472 |
| N1 | −0.437860 | −0.497411 | −0.4092 |
| H1 | 0.336481 | 0.331128 | 0.3234 |
| C2 | 0.290652 | 0.421687 | 0.2284 |
| H2 | 0.149789 | 0.144911 | 0.1571 |
| N3 | −0.598230 | −0.715215 | −0.5392 |
| C4 | 0.254365 | 0.429337 | 0.3033 |
| RMSD | | 0.081416 | 0.0359 |

**2-amino-deoxyribosylpurine nucleotide (D2AP)**

| Atom | This work | Case[a] | Remington et al [33] |
|---|---|---|---|
| C1' | 0.056130 | 0.043100 | – |
| H1' | 0.160136 | 0.183800 | – |
| N9 | −0.012219 | −0.116300 | −0.047936 |
| C8 | 0.181055 | 0.261000 | 0.150546 |
| H8 | 0.182007 | 0.131000 | 0.165748 |
| N7 | −0.640042 | −0.635000 | −0.598370 |
| C5 | 0.153173 | 0.090000 | 0.156676 |
| C6 | 0.200186 | 0.234000 | 0.157719 |
| H6 | 0.152956 | 0.137000 | 0.150049 |
| N1 | −0.751904 | −0.748000 | −0.674915 |
| C2 | 1.012880 | 0.981000 | 0.879792 |
| N2 | −1.009343 | −0.961000 | −0.930086 |
| H21 | 0.425372 | 0.400500 | 0.394869 |
| H22 | 0.425372 | 0.400500 | 0.394869 |
| N3 | −0.758104 | −0.758000 | −0.682870 |
| C4 | 0.343944 | 0.478000 | 0.378610 |
| RMSD | | 0.055083 | 0.056490 |

[a] Unpublished data from Dr. David Case at http://amber.manchester.ac.uk/

**2,6-diamino-ribosylpurine nucleotide (DDAP) = 2-amino-adenylate (D2AA)**

| Atom | This work | Lankas et al [34] |
|---|---|---|
| C1' | 0.022913 | 0.0431 |
| H1' | 0.171954 | 0.1838 |
| N9 | 0.079158 | 0.025808 |
| C8 | 0.104267 | 0.118767 |
| H8 | 0.190196 | 0.189502 |
| N7 | −0.613923 | −0.648666 |
| C5 | 0.160356 | 0.32085 |
| C6 | 0.551922 | 0.399885 |
| N6 | −0.842037 | −0.828851 |
| H61 | 0.401201 | 0.408105 |
| H62 | 0.401201 | 0.408105 |
| N1 | −0.759932 | −0.662095 |
| C2 | 0.901869 | 0.82052 |
| N2 | −0.930769 | −0.898822 |
| H21 | 0.404765 | 0.382157 |
| H22 | 0.404765 | 0.382157 |
| N3 | −0.732276 | −0.669702 |
| C4 | 0.205970 | 0.14698 |
| RMSD | | 0.0665 |

**7-deaza-deoxyguanidylate (D7AG)**

| Atom | This work |
|---|---|
| C1' | −0.018878 |
| H1' | 0.185889 |
| N9 | 0.140071 |
| C8 | −0.232052 |
| H8 | 0.243381 |
| C7 | −0.424411 |
| H7 | 0.236499 |
| C5 | −0.030296 |
| C6 | 0.515155 |
| O6 | −0.588116 |
| N1 | −0.474387 |
| H1 | 0.335679 |
| C2 | 0.792251 |
| N2 | −0.980022 |
| H21 | 0.434283 |
| H22 | 0.434283 |
| N3 | −0.699623 |
| C4 | 0.251894 |

**Iso-deoxyguanidylate (iDG)**

| Atom | This work | Bachmann et al [30] |
|---|---|---|
| C1' | 0.070203 | 0.035863 |
| H1' | 0.163308 | 0.174553 |
| N9 | 0.023544 | −0.588162 |
| C8 | 0.142151 | 0.326728 |
| H8 | 0.187259 | 0.148758 |
| N7 | −0.623484 | −0.616939 |
| C5 | 0.219531 | 0.070026 |
| C6 | 0.340225 | 0.585878 |
| N6 | −0.820639 | −0.868264 |
| H61 | 0.420274 | 0.471138 |
| H62 | 0.420274 | 0.452016 |
| N1 | −0.420543 | −0.751656 |
| H1 | 0.337469 | 0.455052 |
| C2 | 0.770406 | 1.031195 |
| O2 | −0.660974 | −0.647017 |
| N3 | −0.688186 | −0.867736 |
| C4 | 0.240781 | 0.712059 |
| RMSD | | 0.235853 |

**5-methyl-iso-deoxycytidylate (D5MiC)**

| Atom | This work |
|---|---|
| C1' | 0.151775 |
| H1' | 0.106839 |
| N1 | −0.066254 |
| C6 | −0.271426 |
| H6 | 0.264632 |
| C5 | −0.019659 |
| C5M | −0.292265 |
| H71 | 0.089458 |
| H72 | 0.089458 |
| H73 | 0.089458 |
| C4 | 0.761736 |
| O4 | −0.621559 |
| N3 | −0.743440 |
| C2 | 0.674113 |
| N2 | −0.920754 |
| H21 | 0.414744 |
| H22 | 0.414744 |

**Table S6.** Perturbation of global fitting on RESP-fitted charges.

RESP-fitted charges were computed for the following residues as a global fit with the four canonical bases as described in the text. In one case (“9+4”), all nine canonical bases were fitted simultaneously with the canonical base set. In the other case (“1+4”), only the shown residue was fitted with the canonical base set. All other settings were identical. Values are shown for the variable atoms (nucleobase and C1'/H1') at full single precision. The shared atoms are per parm94.

**Deoxyinosinate (DI)**

| Atom | “9+4” | “1+4” |
|---|---|---|
| C1' | 0.083144 | 0.083144 |
| H1' | 0.157553 | 0.157553 |
| N9 | −0.009820 | −0.009820 |
| C8 | 0.109175 | 0.109175 |
| H8 | 0.205646 | 0.205646 |
| N7 | −0.577677 | −0.577677 |
| C5 | 0.209141 | 0.209141 |
| C6 | 0.522966 | 0.522966 |
| O6 | −0.573726 | −0.573726 |
| N1 | −0.437860 | −0.437860 |
| H1 | 0.336481 | 0.336481 |
| C2 | 0.290652 | 0.290652 |
| H2 | 0.149789 | 0.149789 |
| N3 | −0.598230 | −0.598230 |
| C4 | 0.254365 | 0.254365 |
| RMSD | | 0.000000 |

**2-amino-deoxyribosylpurine nucleotide (D2AP)**

| Atom | “9+4” | “1+4” |
|---|---|---|
| C1' | 0.056130 | 0.056134 |
| H1' | 0.160136 | 0.160136 |
| N9 | −0.012219 | −0.012222 |
| C8 | 0.181055 | 0.181055 |
| H8 | 0.182007 | 0.182006 |
| N7 | −0.640042 | −0.640035 |
| C5 | 0.153173 | 0.153155 |
| C6 | 0.200186 | 0.200204 |
| H6 | 0.152956 | 0.152952 |
| N1 | −0.751904 | −0.751913 |
| C2 | 1.012880 | 1.012888 |
| N2 | −1.009343 | −1.009349 |
| H21 | 0.425372 | 0.425374 |
| H22 | 0.425372 | 0.425374 |
| N3 | −0.758104 | −0.758110 |
| C4 | 0.343944 | 0.343951 |
| RMSD | | 0.000008 |

**5-methyl-deoxycytidylate (D5MC)**

| Atom | "9+4" | "1+4" |
|---|---|---|
| C1' | −0.008195 | −0.008193 |
| H1' | 0.205216 | 0.205216 |
| N1 | −0.062430 | −0.062444 |
| C6 | −0.160421 | −0.160403 |
| H6 | 0.261137 | 0.261133 |
| C5 | −0.069175 | −0.069181 |
| C5M | −0.244302 | −0.244313 |
| H71 | 0.075076 | 0.075079 |
| H72 | 0.075076 | 0.075079 |
| H73 | 0.075076 | 0.075079 |
| C4 | 0.617608 | 0.617612 |
| N4 | −0.843162 | −0.843162 |
| H41 | 0.397341 | 0.397341 |
| H42 | 0.397341 | 0.397341 |
| N3 | −0.723926 | −0.723930 |
| C2 | 0.785741 | 0.785748 |
| O2 | −0.656402 | −0.656403 |
| RMSD | | 0.000007 |

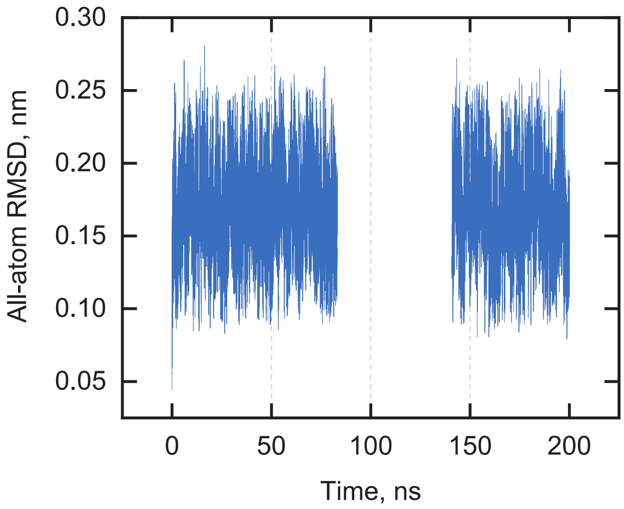


**Figure S1. Equilibration of the DNA hairpin in unrestrained MD simulations.** RMSD of all atomic positions in the hairpin following 200 ns of unrestrained simulation. The trajectory for the d(2AP):dT hairpin is shown; others are similar. Dashes delimit 50-ns blocks of trajectories for averaging of replicate structures starting at 50 ns.
